# A Man With Two Pacemakers: The Mystery of the Electrocardiogram

**DOI:** 10.1002/ccr3.70338

**Published:** 2025-03-27

**Authors:** Yingchun Hu, Xiaoyu Chen, Hui Huang, Weiming Luo, Yisheng Zhou, Xingkao Chen, Guoping Liu, Hanping Zhang

**Affiliations:** ^1^ Department of Cardiology Guangzhou Development District Hospital Guangzhou Guangdong China; ^2^ Department of Nephrology, Rheumatism and Immunology Chongqing Jiulongpo People's Hospital Chongqing China

**Keywords:** battery depletion, electrocardiogram, his bundle pacing, pacemaker, pacing spikes

## Abstract

Our findings reported a confusing but interesting clinical practice. In our study, three spike signals are observed during stable pacing periods, with fixed spikes occurring during the AV delay at 180 ms intervals, excluding other operation types such as CRT and His bundle pacing.

**Abbreviations**
AV: atrioventricular
bpm: beat per minute
CRT: cardiac resynchronization therapy
DDD: dual mode, dual sensing, dual pacing
ECG: electrocardiogram
NT‐ProBNP: N‐terminal pro‐brain natriuretic peptide
VAT: ventricular pacing, atrial sensing, tracking
VOO: ventricular pacing, no sensing

## 1 Introduction

The increasing growth in the elderly population and expanding indications for pacemaker use have led to a progressive rise in the number of pacemaker implants. As the number of pacemaker implants continues to grow, the demand for pacemaker replacements also increases. Therefore, regular follow‐up and timely detection of pacemaker issues are particularly important. Pacemaker interrogation is an important process during the patients with pacemaker implantation, which relates to a life‐threatening matter. The consequences associated with pacemakers include various items such as output malfunction, capture failure, extended RR intervals, or even cardiac arrest. It is rare that three spike signals are observed during the AV delay, which is more commonly seen in cases of CRT, His bundle pacing, and left bundle branch pacing.

## 2 Case Presentation

**FIGURE 1:**
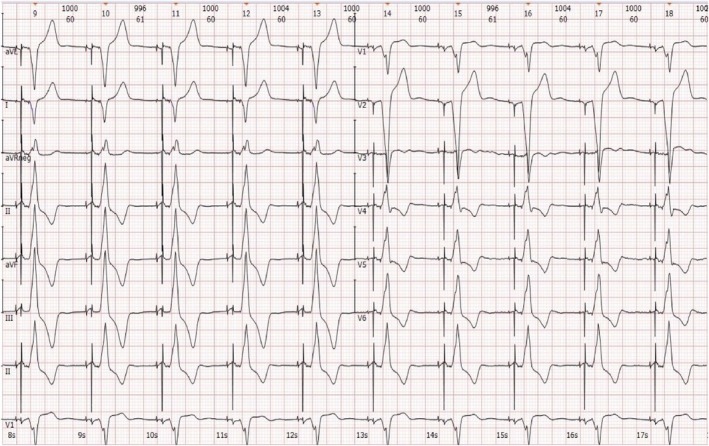
Cabrera sequence recording. The above pacing ECG shows sequential capture of the atrium and ventricle, with dominant spikes at the beginning of the P wave and an aberrant QRS complex, maintaining a basic cycle of 1000 ms. The ECG reveals right atrial appendage pacing with a positive P wave in leads I, II, III, and aVF, a negative P wave in lead aVR, and a biphasic P wave in lead V1, with a PtfV1 of 0.05 mm/s. The ventricular electrode is positioned in the right ventricular outflow tract, indicated by a left bundle branch morphology in lead V1, a QS complex in leads I and aVL, and a tall R wave in leads II, III, and aVF. Additionally, three spike signals are observed during stable pacing periods, with fixed spikes occurring during the AV delay at 180 ms intervals, excluding other operation types such as CRT and His bundle pacing. Upon encountering this ECG, a pacemaker interrogation was performed immediately. AV, atrioventricular; CRT, cardiac resynchronization therapy; ECG, electrocardiogram.

**FIGURE 2:**
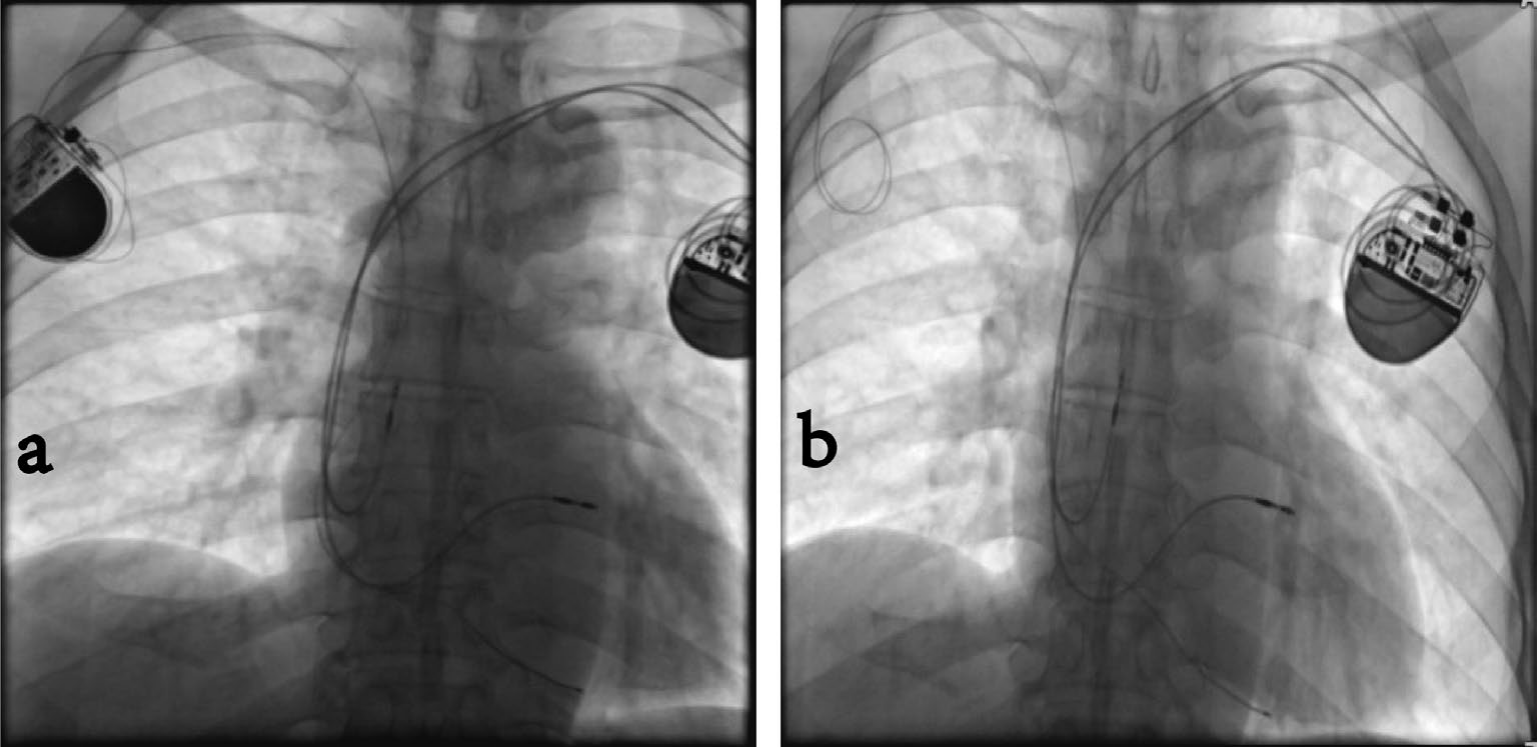
(a, b) X‐ray contrast images before and after pacemaker extraction. The two X‐rays above show the contrast images taken before and after pacemaker extraction. The left image (a) displays two pacemaker generators positioned below the bilateral clavicles. The pacemaker on the right side is a 15‐year‐old single‐chamber pacemaker, with its electrode lead located at the right ventricular apex. The pacemaker on the left side is a 1‐week‐old dual‐chamber pacemaker with pacing electrodes situated in the right atrial appendage and the proximal septum. The right image (b) shows the single‐chamber pacemaker generator extraction with the passive electrode remaining in place.

A 51‐year‐old man with a permanent pacemaker implanted 15 years ago for sick sinus syndrome presented to the emergency department after experiencing a week of palpitations. He had not regularly followed up on the pacemaker after the operation. Two weeks ago, he had a dual‐chamber pacemaker implanted due to battery depletion in another hospital. A bedside electrocardiogram (ECG) and chest X‐ray were obtained (Figures 1 and 2a).

**FIGURE 3:**
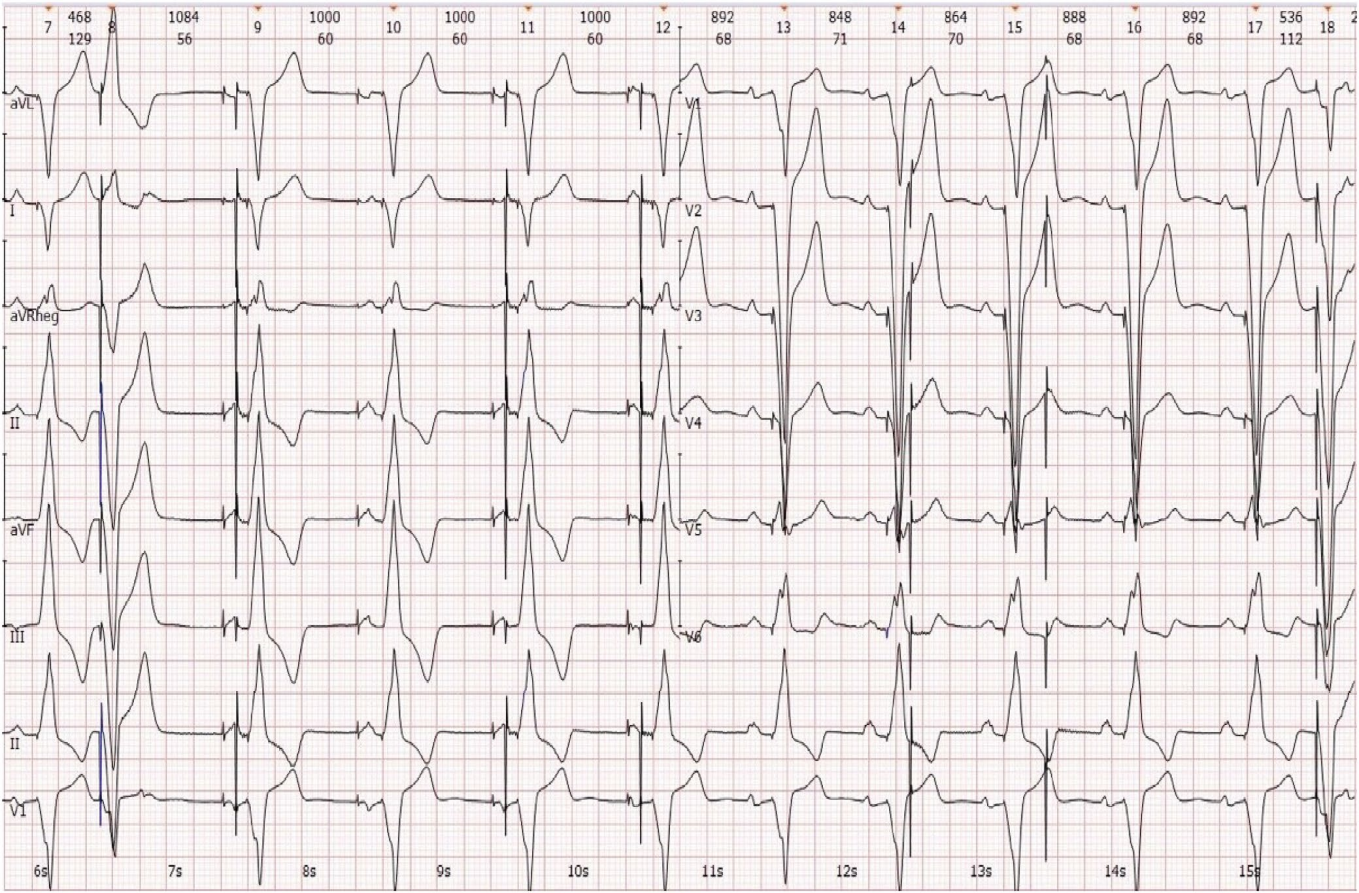
Cabrera sequence recording. The above ECG characterized by two pacemakers indicates the following: Firstly, the patient has a dual‐chamber pacemaker operating in DDD or VAT mode, with both sensing and pacing functions being normal. Secondly, the pacemaker is in the stage of battery depletion and has automatically switched to VOO mode. Additionally, the pacing rate alternates between 30 bpm and 60 bpm, with a few unipolar pacing spikes successfully capturing the ventricle (as seen in the second and last aberrant QRS complexes). Lastly, the QS complexes in the inferior and precordial leads, as well as the R wave in leads I and aVL, align with the pacing characteristics of the right ventricular apex. Meanwhile, a chest X‐ray confirms the anatomical locations of the electrodes. This ECG demonstrates the characteristics of both pacemakers, leading us to recommend extracting the old pacemaker generator (Figure 2b). DDD, dual mode, dual sensing, dual pacing; ECG, electrocardiogram; VAT, ventricular pacing, atrial sensing, tracking; bpm, beats per minute; VOO, ventricular pacing, no sensing.

**FIGURE 4:**
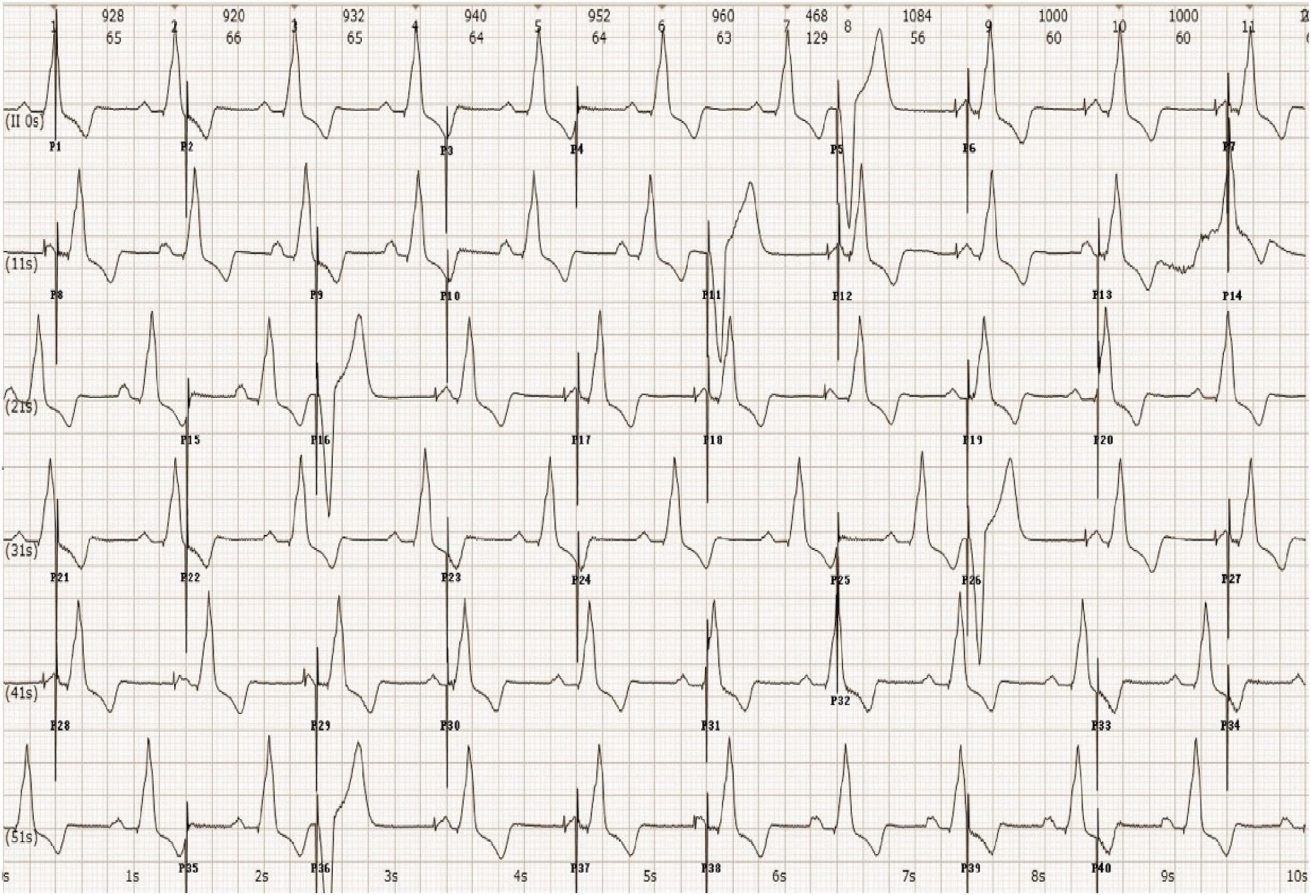
Prolonged recording of lead II for 60 s. The above ECG characterized by two pacemakers indicates the following: Firstly, the patient has a dual‐chamber pacemaker operating normally in DDD or VAT mode. Secondly, the pacemaker is in the stage of battery depletion and has automatically switched to VOO mode. Additionally, the pacing rate alternates between 30 and 60 bpm, with intermittent ventricle capture observed. In summary, the ventricle capture is not related to whether the pacing spikes are odd or even, thus eliminating the possibility that the pacemaker is reserving power for a minute due to battery depletion. Instead, ventricular capture is associated with the supernormal phase of myocardial activity, which can respond to subthreshold stimuli. The ventricular supernormal interval is determined to range from 440 ms to 520 ms. DDD, dual mode, dual sensing, dual pacing; ECG, electrocardiogram; VAT, ventricular pacing, atrial sensing, tracking; bpm, beats per minute; VOO, ventricular pacing, no sensing.

Upon arrival at the emergency department, his heart rate was 60 beats per minute (bpm), respiratory rate was 21 bpm, blood pressure was 123/61 mmHg, and oxygen saturation was 98% on room air, with a normal body temperature. The patient underwent a comprehensive cardiovascular, pulmonary, and neurological workup, which excluded structural diseases. The levels of electrolytes, glucose, cardiac enzymes, N‐terminal pro‐brain natriuretic peptide (NT‐ProBNP), coagulation tests, D‐dimer, renal and liver functions, and complete blood count were all normal. Despite this, the patient repeatedly complained of palpitations, prompting the performance of two additional bedside ECGs (Figures 3 and 4).

## 3 Methods

We conducted the differential diagnoses including CRT, His bundle pacing, and left bundle branch pacing. In order to obtain an exact diagnosis, we arranged for this patient a bedside chest X‐ray. From the chest X‐ray, we failed to find a relevant pacing electrode placed in the field of the left ventricle, His bundle, and left bundle branch. The pacemaker on the right side is a 15‐year‐old single‐chamber pacemaker, with its electrode lead located at the right ventricular apex (Figure 2a). The pacemaker on the left side is a 1‐week‐old dual‐chamber pacemaker with pacing electrodes situated in the right atrial appendage and the proximal septum (Figure 2a).

Moreover, three spike signals are observed during stable pacing periods, with fixed spikes occurring during the AV delay at 180 ms intervals, excluding other operation types such as CRT and His bundle pacing.

## 4 Conclusion

The patient experienced constant three spike signals during the AV delay purely by coincidence. The pacing spikes generated by a pacemaker with battery depletion exhibit alternation between long and short intervals, resulting in a 3:2 spike output pattern.

## 5 Discussion

The increasing growth in the elderly population and expanding indications for pacemaker use have led to a progressive rise in the number of pacemaker implants since the invention of the first implantable pacemaker in the 1950s. As is well known, the typical lifespan of a pacemaker is generally 6 to 10 years, depending on factors such as the percentage of pacing and sensing, output settings, frequency parameters, and impedance. Each pacemaker has indicators that suggest the recommended replacement time, including the elective replacement indicator, pacing mode, and changes in magnetic frequency. Ignoring these indicators or failing to follow up regularly can lead to serious consequences for patients, such as output malfunction, capture failure, extended RR intervals, or even cardiac arrest. As the number of pacemaker implants continues to grow, the demand for pacemaker replacements also increases. Therefore, regular follow‐up and timely detection of pacemaker issues are particularly important.

Our study observed three spike signals during the AV delay (Figure 1), which are more commonly seen in cases of CRT, His bundle pacing [1], and left bundle branch pacing. In these cases, dislocation or an increase in threshold can lead to the pacing failure of the left ventricular lead, His bundle pacing lead, or left bundle branch pacing. As a result, the spare right ventricular pacing electrode may capture the myocardium. However, transient three‐spike signals can also occur with advanced pacemaker functions, such as atrial or ventricular threshold examinations. Considering the medical history, chest X‐ray examinations, and repeated ECGs of the patient, it was determined that the patient, who had two pacemakers implanted, experienced constant three spike signals during the AV delay purely by coincidence.

The pacing spikes generated by a pacemaker with battery depletion exhibit alternation between long and short intervals, resulting in a 3:2 spike output pattern. Most of these spikes result in loss of capture, which can be attributed to spikes occurring during the refractory period of the ventricular myocardium or during the diastolic period. In the former case, the spikes occur on the QRS complex, ST segment, or T wave, while in the latter case, the spikes represent a subthreshold stimulus. Our findings indicate that pacing capture is not influenced by whether the spike signals are even or odd, thereby excluding transient power contributions from an exhausted battery. Additionally, our report highlights a supernormal period at the end of myocardium repolarization, which is considered a subthreshold stimulus that can excite myocardial tissue.

Previous study by Kazatsker et al. [2] had also reported a man with two pacemakers in the chest. But in their report, one of the pacemakers implanted for cardiac pacing and the other one was for brain stimulation as a treatment for severe Parkinson's disease, evidenced by leads oriented toward the neck. In our study, the two pacemakers were both for heart disorders, with the electrodes located in the heart cavity, and we further made a detailed analysis according to the interrogation information.

A recent study suggests that syncope caused by pacemaker malfunction is a rare cause of hospitalization [3]. From our study, we learn that operators must exercise caution when dealing with patients requiring replacement due to pacemaker power depletion. Rushed decisions can lead to adverse effects. For instance, although the patient in our study avoided severe consequences, he faced economic burdens and mental distress.

## Author Contributions

**Yingchun Hu:** conceptualization, resources, supervision, writing – original draft. **Xiaoyu Chen:** data curation, software, validation, writing – review and editing. **Hui Huang:** formal analysis, supervision, validation, visualization. **Weiming Luo:** funding acquisition, resources, software, visualization. **Yisheng Zhou:** investigation, validation, writing – original draft. **Xingkao Chen:** methodology, software, writing – review and editing. **Guoping Liu:** project administration, resources, validation. **Hanping Zhang:** resources, supervision, writing – original draft, writing – review and editing.

## Ethics Statement

This article does not contain any studies with human participants and/or animals.

## Consent

Published with written consents of the patient.

## Conflicts of Interest

The authors declare no conflicts of interest.

## Supporting information


Data S1.

## Data Availability

The data that support the findings of this study are available from the corresponding author upon reasonable request.
