# Supplementary material for: A Man With Two Pacemakers: The Mystery of the Electrocardiogram
Source: Clin Case Rep. 2025 Mar 27;13(4):e70338. doi: 10.1002/ccr3.70338 (PMC11947422; doi:10.1002/ccr3.70338)
Supplement: Supplementary file 1 — Data S1. [file CCR3-13-e70338-s001.pdf]

Bullet Edits

EDITORIAL CERTIFICATE

This document certifies that the paper listed below was edited and proofread for proper English language, grammar, punctuation, spelling, and overall style by one or more than one highly qualified native speakers at Bullet Edits. All of the suggested amendments were tracked with the Microsoft Word "Track Changes" feature. Therefore, the author had the option to reject or accept each change individually.

Manuscript Title:

A man with two pacemakers: the mystery of the electrocardiogram

Anti-counterfeiting Code:

ea5629d8eb9507af71d2f0d257903ccd

Date Issued:

2024-08-05 14:52:20

Bullet Edits is a registered company headquartered in the UK with a global presence. We offer a range of editing, proofreading services to authors. Our Ph.D. editors are all native English speakers from the USA and UK. Authors who work with Bullet Edits are guaranteed excellent language quality and timely delivery.

Address: BULLET EDITS LIMITED, 85 Great Portland Street, London,UK
Tel: UK. (+44)20457 70286 / US.(+1)312-313-9179
Web: www.bulletedits.cn/Email: info@bulletedits.cn
VAT: GB 378 9316 43

BULLET EDITS LIMITED ★ COMPANY NO. 13345261 SEAL ★ ENGLAND AND WALES
